# Community environment and COVID-19 related stress among older adults with disabilities in Taiwan

**DOI:** 10.1093/geroni/igaa057.3429

**Published:** 2020-12-16

**Authors:** Meng-Hsuan Yu, Shiau-Fang Chao

**Affiliations:** 1 National Taiwan University, Taipei City, Taipei, Taiwan (Republic of China); 2 National Taiwan University, Taipei City, Taiwan (Republic of China)

## Abstract

The outbreak of the COVID-19 pandemic at the beginning of 2020 forced many countries to implement social distancing policies such as the suspension of activities and gathering. Taiwan is the leading country which took active epidemic prevention measures in local communities, including closing the community centers and programs for older adults. Older adults with chronic health conditions are particularly vulnerable to the COVID-19 pandemic because they have disproportionally been affected by it. This study examined the associations between community environment and COVID-19 related stress of community-dwelling older adults with disabilities. Data were collected from a sample of 547 community-dwelling older adults aged 65 and over with disabilities in Taiwan between April and July, 2020. Multiple Regression Analysis was applied to test the hypothesized relationships. The analytic results suggested that: First, participants who were younger and with better cognitive functioning had higher levels of COVID-19 related stress. Second, as the confirmed case number dropped by month, participants interviewed in the later months expressed lower levels of COVID-19 related stress. Third, older adults who perceived more obstacles in their environment reported higher levels of COVID-19 related stress. In conclusion, although restrictions during the pandemic is inevitable to secure the safety of the public, programs should be designed for older adults with disabilities to remove the obstacles and to make information, policies and services more accessible in the communities to mitigate their COVID-19 related stress.

